# Susceptibility to Tuberculosis Is Associated With PI3K-Dependent Increased Mobilization of Neutrophils

**DOI:** 10.3389/fimmu.2018.01669

**Published:** 2018-07-17

**Authors:** Gina R. Leisching

**Affiliations:** DST-NRF Centre of Excellence for Biomedical Tuberculosis Research, South African Medical Research Council Centre for Tuberculosis Research, Division of Molecular Biology and Human Genetics, Faculty of Medicine and Health Sciences, Stellenbosch University, Cape Town, South Africa

**Keywords:** neutrophilia, tuberculosis, susceptibility, PI3-kinase, *Mycobacterium tuberculosis*

## Abstract

Neutrophilia is a condition commonly observed in patients with late-stage tuberculosis, but evidence suggests that increased neutrophil influx begins early after infection in susceptible hosts and functions to promote a nutrient-replete niche that promotes *Mycobacterium tuberculosis* survival and persistence. As the disease progresses, an increase in the number of neutrophil-like cells is observed, all of which exhibit characteristics associated with (i) phenotypic and biochemical features of immaturity, (ii) the inability to activate T-cells, (iii) hyper-inflammation, and (iv) prolonged survival. Transcriptomics reveal a common set of molecules associated with the PI3–Kinase pathway that are dysregulated in patients with active tuberculosis. Closer inspection of their individual biological roles reveal their ability to modulate the IL-17/G–CSF axis, induce leukocyte receptor activation, and regulate apoptosis and motility. This review draws attention to neutrophil hyper-reactivity as a driving force for both the establishment and progression of tuberculosis disease in susceptible individuals.

## Introduction

Evidence to date suggests a link between the advanced stages of tuberculosis and neutrophilia. In order for neutrophils to accumulate within the lung, an orderly procession of cell–endothelial interaction, transcellular or pericellular transmigration, and some degree of enhanced survival or defective egress is required. Overall, evidence points toward a disruption in some of these key events during advanced tuberculosis disease. Multiple studies suggest that neutrophils play a protective role in the prevention of tuberculosis and report antimycobacterial effects in a number of *in vivo* studies (1–3). It has also been shown that neutrophil-derived bactericidal molecules kill *Mycobacterium tuberculosis in vitro* (4, 5). Human studies further reveal that higher neutrophil counts are protective against early tuberculosis infection (6), thus during the initial stages infection of infection, neutrophils play a protective role; however, a pathogenic role for neutrophils during the late stages of tuberculosis has been proposed (7). Neutrophilia has been assigned as a predictor of disease progression, pulmonary destruction, and even death (6, 8–13). The neutrophil/lymphocyte ratio (NLR) is able to distinguish tuberculosis patients from tuberculin skin test-positive healthy contacts (8) and predict pulmonary tuberculosis retreatment (14). Additionally, the significance of neutrophils during active tuberculosis is highlighted by the fact that the blood transcriptional signature of tuberculosis is neutrophil-driven and effectively distinguishes tuberculosis from other inflammatory diseases (15). Neutrophils have, however, been shown to mediate an early inflammatory response that is critical for controlling *M. tb* infection. Thus, in the very early stages, evidence suggests that in resistant individuals, neutrophils are likely to control *M. tb* infection in a more effective manner than in susceptible individuals. It should also be noted, however, that not all patients with tuberculosis exhibit neutrophilia. In those that do, it is observed that various sub-sets of neutrophils exist with differing phenotypic characteristics.

**Figure 1:**
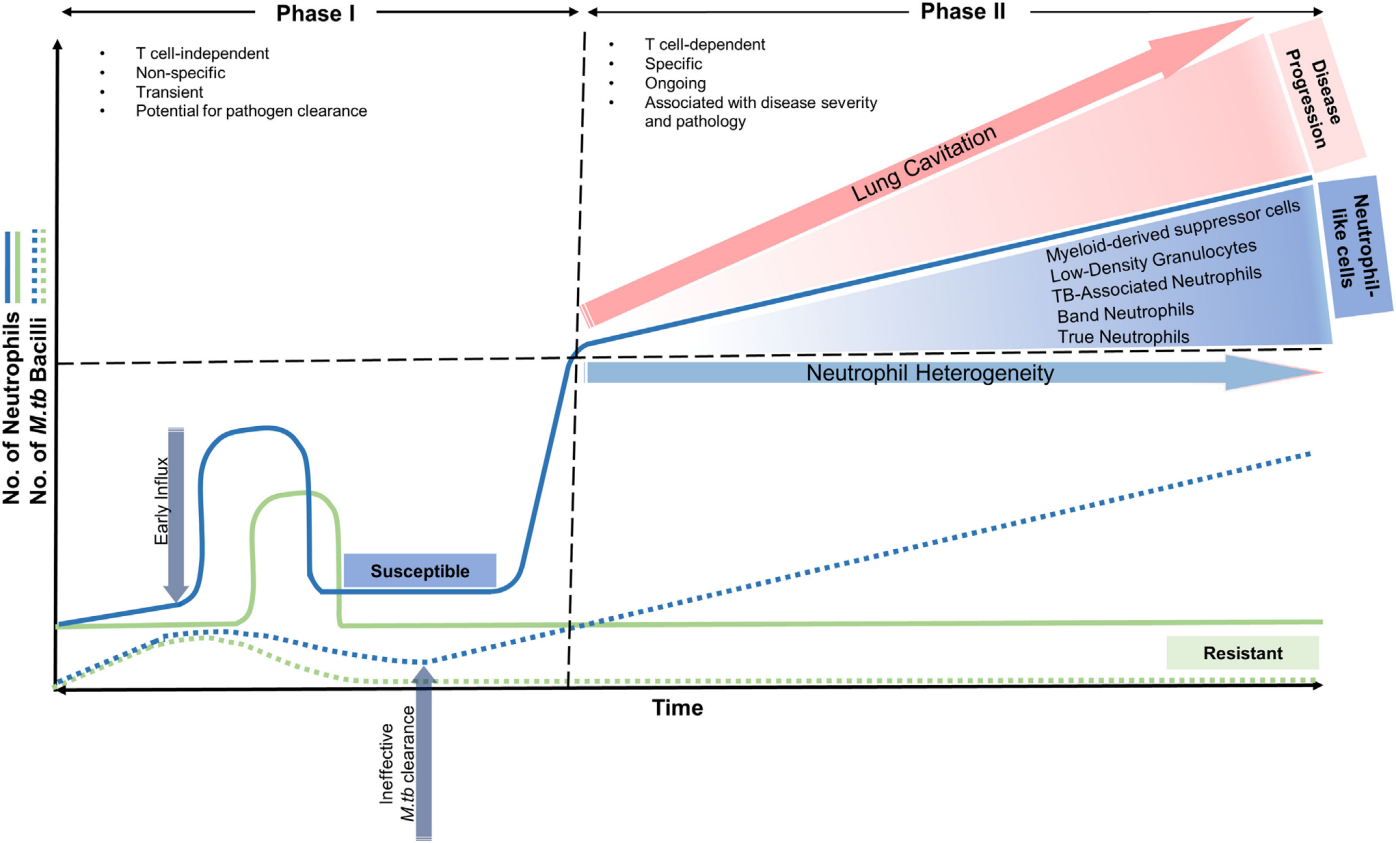
Neutrophil recruitment into the lung in resistant and susceptible individuals over time in early and advanced stages of *Mycobacterium tuberculosis* infection. In resistant individuals, neutrophil influx occurs soon after infection, where along with macrophages, aid in removing *M. tuberculosis* effectively. Neutrophil numbers return to baseline and a minimal effect on the host is observed. In susceptible individuals, phase I begins soon after infection and is characterized as T cell-independent, non-specific, and transient with the potential for pathogen clearance. Phase II is characterized as T cell-dependant, specific, and ongoing and is associated with disease severity and pathology. During this stage, and as the disease progresses, an increase in the number of neutrophil-like cells (NLC) circulating in the blood and those from lung tissue are observed. They present as immature or undifferentiated precursor cells in the form of myeloid-derived suppressor cells (MDSC), tuberculosis-associated neutrophils (TBAN), low-density neutrophils, and band neutrophils. The second phase of exacerbated neutrophil influx is also associated with lung cavitation and disease progression.

Neutrophil influx to the lung after *M. tb* infection occurs in two phases in susceptible individuals (Figure 1): the first phase begins soon after infection and is characterized as T cell-independent, non-specific, and transient with the potential for pathogen clearance (16–18). The second phase is characterized as T cell-dependant, specific, and ongoing and is associated with disease severity and pathology (13, 19, 20). During this stage, and as the disease progresses, an increase in the number of neutrophil-like cells (NLC) circulating in the blood and those from lung tissue are observed. They present as immature or undifferentiated precursor cells in the form of myeloid-derived suppressor cells (MDSC) (21–23), tuberculosis-associated neutrophils (TBAN) (23), low-density neutrophils (24), and band neutrophils (20). The role of these precursor/immature neutrophils at the infection site generates a number of questions with regards to their phagocytic abilities. In other words, they are able to phagocytose *M. tb*, but fail to eliminate the bacteria, or they do not phagocytose mycobacteria, but rather associate with T-cells (25). The mechanisms underlying this second phase of exacerbated neutrophil infiltration into the lungs is largely unknown. During acute infection, in immune-competent individuals, neutrophil chemotaxis is controlled by a fine balance between mitogen-activated protein kinase (p38 MAPK) (26) and phosphatidylinositol 3-kinase (PI3-K) signaling. Briefly, when neutrophils are activated by chemoattractants, PI3-K phosphorylates phosphatidylinositol 4,5-bisphosphate (PIP2) into phosphatidylinositol (3,4,5)-trisphosphate (PIP3), which promotes their directional migration (27). Any perturbations in the expression of these genes coding for components of PI3-K pathway, including its regulators may disrupt neutrophil trafficking and lead to neutrophil hyperreactivity (28).

In this review, attention is drawn to the link between neutrophil hyperreactivity and the progression of tuberculosis disease as a characteristic of the susceptible host. Further, common characteristics of these neutrophils across mouse and human studies are highlighted. A closer look at transcriptomic studies in human and mouse models of tuberculosis reveals altered expression levels of members of the PI3-kinase family and support not only a possible mechanism underpinning neutrophil hyperreactivity but may also explain the ineffectiveness of the adaptive immune response observed during tuberculosis. Using the latest evidence, an attempt is made to elucidate whether enhanced neutrophil trafficking is a function of uncontrolled inflammation or whether specific signals drive neutrophil production during tuberculosis. These mechanisms span over a multitude of cytokines, leukotrienes, complement factors, adaptor molecules, and receptors whose expression may be found ultimately to be dysregulated as a factor of host genetic aberrations.

## Susceptibility to Tuberculosis is Associated with a Higher Abundance, Increased Migratory Capacity, and Heterogeneity of Neutrophils

Immune hyperreactivity underlies the development of tuberculosis disease and is characterized by an over secretion of proinflammatory cytokines and chemokines, exacerbated T cell responses, and increased neutrophil infiltration in the lungs (20). Neutrophils are the most abundant immune cell found in the bronchoalveolar lavage (BAL) and sputum of active pulmonary tuberculosis patients, and in the lungs are second only to lymphocytes (9).

The early immune response to *M. tb* infection is not possible to assess in human subjects; however, mice studies of susceptibility have been able to capture these early immune events with regard to the response of granulocytes soon after mycobacterial infection. Common characteristics of neutrophils from susceptible mouse strains indicate that they have an intrinsically higher migration activity, which does not depend on the presence of an infectious stimulus (29). It has been observed that in both I/St and DBA/2 susceptible mouse strains, there is a significant influx of neutrophils early on after infection when compared to the resistant strains (29, 30). Since these mice are bred for susceptibility, this cause of this response is attributed to host-based genetics that are independent of the infectious agent. In both mice and humans, *M. tb* is still not cleared effectively in susceptible hosts, despite the increased number of neutrophils at the infection site. Up until now, it was unknown whether the abundance of neutrophils just days after infection promote *M. tb* survival. Recent work suggests that this hyperreactive influx early on primes the affected tissue for mycobacterial clinical persistence: it was demonstrated that granulocytic inflammation generates a nutrient-replete niche that supports *M. tb* growth, specifically *M. tb* that grow in association with neutrophils encounter a more hospitable environment that is replete with micronutrients, such as iron and lipid carbon sources (13). This finding proposes that this early influx may promote *M. tb* replication and, therefore, disease progression. A study in patients with recent tuberculosis (patients had not yet received anti-tuberculosis therapy), observed neutrophilia as a common characteristic among patients and a correlation between pulmonary pathology and band neutrophils was confirmed (20). Generally, elevated band counts (bandemia) suggest serious bacterial infection; however, it is not suggested as parameter for the diagnosis of infection (31). In the context of early tuberculosis, it may, therefore, be an indication of hyperactivity of the granulocyte response to infection in susceptible individuals. It may, therefore, be inferred that the main difference in the early neutrophil response to *M. tb* infection between susceptible and resistant individuals is (i) the number of neutrophils at the site of infection early post-infection, (ii) the ability of neutrophils to phagocytose, but not eliminate *M. tb* despite these high numbers and finally, (iii) the inability of these neutrophils to effectively communicate with other immune cells to activate an efficient adaptive immune response (discussed below).

In advanced tuberculosis, neutrophil abundance is an overwhelming feature associated with disease severity. In order to accommodate this influx, other cells, such as the endothelium are also primed as a consequence. It was observed that the endothelial mediators of neutrophil migration, namely l-selectin, E-selectin, and ICAM-1 are upregulated in active tuberculosis (32), which correlates with the observation that neutrophils are present at sites of active mycobacterial disease (9, 33, 34). Neutrophil trafficking seems to be in-part, associated with the expression of programed death-1 (PDL-1), since PD-1 or PDL-1 knockout mice show exaggerated pathology and excess neutrophil influx (35). In the case of active tuberculosis, however, PDL-1 is overexpressed by neutrophils (36), which suggests that either PDL-1 excess or deficiency may result in neutrophil-associated pathology that is linked to exacerbated influx (18). This effect is likely to be indirect since PDL-1 expression has been shown to inhibit T-cell effector functions during tuberculosis disease (37) and may explain the delayed accumulation of T-cell responses within the lung that (i) diminish communication between the adaptive and innate immune systems and (ii) prevent clearance of *M. tb*, leading to latency (38). An interesting concept of “adaptive neutrophils” was introduced through a mouse study of *M. tb* infection where the authors describe that upon the onset of the adaptive immune response, an influx of neutrophils, together with T-cells were recruited to the lung (25). It is interesting to note that these neutrophils did not associate with the bacteria, but rather interacted closely with T-cells. This non-phagocytic nature is unusual for neutrophils and suggests an altered neutrophil phenotype exists during established disease. The emerging concept of neutrophil heterogeneity that is observed in the blood of tuberculosis patients and mouse studies of *M. tb* infection supports this observation. Both human and mouse models of late-stage infection indicate that the neutrophil population is composed of different subsets that differ in their functional properties, phenotype, and levels of maturity. It is now known that the commensal microbiota regulate steady-state granulopoeisis by regulating G-CSF production (39), and it has been hypothesized that since neutrophils play a prominent role in containing infections, microbes may dictate multiple aspects of neutrophil biology. A study exemplifying this was based on analyzing the susceptibility of mice preconditioned by mild or severe systemic inflammatory response syndrome (SIRS) to *Staphylococcus aureus* infection (40). Neutrophils from mice with severe SIRS produce IL-10, are CD49d^neg^ CD11b^hi^ and uniquely express toll-like receptor-7 (TLR7). In contrast, those neutrophils from mice with mild SIRS are resistant to *S. aureus* and produce IL-12, uniquely express TLR5 and 8, and are CD49d^+^. Further, it was demonstrated that these neutrophil subsets activate macrophages with varying polarity *in vitro*, indicating that these subsets may shape environments, which are microbicidal, inflammatory, or immunosuppressive (40). More importantly, the presence of neutrophils with immunosuppressive or hyperinflammatory characteristics may underpin the susceptibility to infections.

Low-density granulocytes (LDG) have recently emerged as one of the subsets found in patients with advanced tuberculosis disease (24). Transcriptomics reveal that these LDGs exhibit signatures, which correlate best with an immature phenotype, which is based on the expression of mRNA associated with granule enzymes and bactericidal proteins (41). In the same study, LDGs exhibited decreased phagocytic activity, enhanced survival, and an increased secretion of pro-inflammatory cytokines. The release of these immature neutrophils from the bone marrow is hypothesized to be a result of stress and that the presence of specific cytokines arrests neutrophil maturation (42). Myeloid-derived suppressor cells (MDSC) are present at higher frequencies in tuberculosis patients when compared to healthy controls and have been shown to have T cell suppressive effects and a higher inflammatory response in coculture (43, 44). In mice, these MDSCs accumulated in tuberculosis-susceptible mouse strains (21, 22) and show low Gr-1/Ly-6G expression, which accompanied a significant decrease in the number of the typical Gr-1/Ly-6G^hi^ neutrophil phenotype (23). Evidence suggests that *M. tb* fosters the generation of MDSCs in bone marrow, and that susceptibility to infection and lethal inflammation is associated with systemic accumulation of MDSCs and the presence of MDSC-like cells in the lung parenchyma (23). Furthermore, it was demonstrated that MDSC accumulation is part of the general inflammatory response to *M. tb* infection and that the dynamics of MDSCs is augmented in a susceptible host developing primary progressive tuberculosis (22). Both clinical and murine studies agree that these cells phagocytose and “harbor” *M. tb*, suppress T-cell function, and accumulate in susceptible mice/infected individuals during the late stages of tuberculosis disease.

In studies describing the presence of these neutrophil-like cells found in advanced tuberculosis, it is evident that the total number of “true” neutrophils is reduced, additionally, these neutrophil-like cells have the following characteristics in common, (i) phenotypic and biochemical features of immaturity, (ii) observed to dampen T-cell responses, (iii) hyperinflammatory, and (iv) prolonged survival (discussed below). Chemotactic factors, as well as intracellular signaling promoting directional migration essentially governs the influx of neutrophils to sights of inflammation; however in tuberculosis, aberrant signaling, whether inherent or induced may contribute to the granulocyte hyperreactivity that is observed in susceptible individuals.

## The Role of PI3-Kinase Signaling in Shaping the Neutrophil Response

Disruptions in PI3-kinase signaling can influence a multitude of cells, especially those in which neutrophils play a dominant role. Increased PI3-K activity, in particular, has been shown to compromise the migratory accuracy of neutrophils, thereby prolonging their tissue transit time, which leads to bystander tissue injury mediated by surface-associated neutrophil proteases (28). Class I PI3-kinases (α, β, γ, and δ) function to promote the directional migration of neutrophils after activation by chemoattractants (45). Recently, a study revealed for the first time that PI3-K α, β, and δ together orchestrate hematopoietic homeostasis, with PI3-K δ being required specifically for leukocyte development (46). It was further elucidated that deficiencies in PI3-Kγ and δ induced neutrophilia by enhancing signaling through the IL-17/G-CSF axis (47). Both γ and δ isoforms have distinct roles in chemokine-induced neutrophil migration (48). The class II PI3-kinases (C2α, C2β, and C2γ) are suggested to play a potential role in agonist-mediated regulation of cellular functions. PI3K-C2β specifically is implicated in the differentiation of hematopoetic cells by retinoic acid (49). Retrospectively, we observe evidence from blood transcriptional signatures in mouse and human studies of tuberculosis disease, which indicate that circulating neutrophils exhibit altered expression in those genes encoding various PI3-K polypetide/substructures and negative regulators of PI3-K. This will be discussed in more detail below.

## *PIK3CD* Expression Modulates the IL-17/G-CSF Axis and Neutrophil Chemotaxis in Tuberculosis

G-CSF drives neutrophil proliferation and differentiation and its secretion is dependent on IL-17 signaling upstream (50). In response to G-CSF, PI3-K δ (*PIK3CD*) produces second messenger signaling molecules, which promote neutrophil movement in response to chemotactic stimuli (51). Interestingly, PI3-K δ has been implicated in intracellular IL-17 signaling (52) and TH17 cell differentiation (53). In a mouse study of tuberculosis susceptibility, total lung RNA from two susceptible mouse strains (DBA/2 and CBA/J) was analyzed 4 weeks post-infection by Affymetrix GeneChip U74 array (30). It was observed that *Pik3cd* was significantly upregulated in whole lung tissue, along with *Il17ra* (IL-17 receptor), both of which were not differentially regulated in lung tissue of resistant mouse strains. In the same study, analysis revealed that transcriptomics of the affected lung tissue correlated with excessive neutrophil influx into the lungs and a bias toward the expression of genes involved in granulocyte pathophysiology. Interestingly, another murine study observed that IL-17RA in non-hematopoietic cells (epithelial and endothelial cells, as well as fibroblasts) is critical for neutrophil recruitment to the lung (25). Evidence also suggests that a decrease/loss in function of PI3-K δ also affects IL-17-mediated neutrophil recruitment. This was exemplified in a murine study where it was observed that a deficiency in expression of PI3-K δ, along with PI3-K γ, enhances the IL-17/G-CSF axis and induces neutrophilia (47). Additionally, it was noted that a deficiency in these sub-units leads to defective B- and T-cell homeostasis, which underscores their role in effective adaptive immune response activation.

The intracellular presence of *M. tb* itself also appears to directly affect the expression of *PIK3CD*. A recent study investigated PI3-K δ signaling in lung lesions of patients with confirmed pulmonary tuberculosis and observed that the expression of PI3-K δ is globally absent throughout tuberculosis granulomas, but present in normal lung tissue from healthy individuals (54). The same study showed that during early infection, *M. tb* disables the genes encoding PI3-K δ/AKT/mTORC1 and MNK regulatory pathways by upregulating a network of microRNAs with sequences targeting the 3′UTRs regions (54). Disruption of this pathway then promotes a tissue-destructive phenotype through MMP-1 upregulation, which promotes lung cavitation. Thus, in granulomas, PI3-K δ is absent; however, the authors do not indicate what the level of expression is in the surrounding lung tissue. This finding is supported by another study that showed that the PI3-K pathway is the key regulator of interleukin-17 driven MMP secretion by airway epithelial cells (55). Evidence from both humans and mice suggests that both a loss and gain in function of *PIK3CD*/PI3-K δ leads to disruption of the G-CSF/IL-17 pathway leading to neutrophil hyperreactivity and ineffective communication between the innate and adaptive immune system.

## *PIK3IP1*, a Negative Regulator PI3-K p110, is Downregulated in Peripheral Leukocytes from Patients with Tuberculosis

Downstream signaling regulation of the PI3-K pathway is central in maintaining intracellular homeostatic conditions and is controlled by a number of regulators. To date, phosphatase and tensin homolog deleted on chromosome 10 (PTEN), inositol polyphosphate 4-phosphatase type II (INPP4B), and SH2 containing inositol 5′-phosphatase (SHIP-1) are well-characterized negative regulators of the PI3-K pathway, all of which act downstream of PI3-K. Recently, PI3-K interacting protein 1 (PIK3IP1) has also been described as a negative regulator of PI3-K and inhibits its activity upstream through proximal allosteric interference with class I PI3K catalytic subunits p110 α, β, and δ (56, 57). To date, there is no information regarding the biological role of PIK3IP1 during tuberculosis infection; however, it may play a part in disruption of the normal life/death time-course of the extravasated neutrophil. Given the propensity of neutrophils to die by “constitutive” apoptosis when utilized for *in vitro* studies, the major challenge in airway research is to determine how and why neutrophils persist in such a large numbers in the lung. Transcriptomics from tuberculosis patients indicate (i) that *PIK3IP1* is part of the transcriptomic signature that discriminates tuberculosis from other inflammatory infections and diseases and (ii) that its expression is downregulated in circulating neutrophils in comparison to healthy controls (15). Its downregulation was further confirmed in two other whole blood transcriptomic-based tuberculosis studies (58, 59). The consequences of PIK3IP1 on PI3K signaling was elucidated in hepatic cell lines where motility and apoptosis was assessed. It was observed that PIK3IP1 expression suppressed motility and promoted apoptosis in isolated mouse hepatocytes in a PI3-K-dependant manner (60). Although a similar effect cannot be assumed to occur in the leukocyte, the regulatory role that PIK3IP1 may play in apoptosis and motility cannot be ignored and requires further investigation.

## *PIK3C2B* is Downregulated in Peripheral Leukocytes from Patients with Tuberculosis

*PIK3C2B* encodes the class II enzymatic isoform C2β, which has a wide tissue distribution and is found to be expressed in lymphocytes. Blood transcriptomics of patients with tuberculosis revealed that that *PIK3C2B* is downregulated when compared to individuals with latent tuberculosis infection (15, 58, 59). Little evidence is available regarding the role of this class II PI3K under normal and disease conditions in the context of the whole organism. Interestingly though, studies of *PI3KC2*β*−*/*−* mice have demonstrated that while *PI3K-C2*β*−/−* mice exhibit no obvious phenotypic abnormalities (61), they exhibit increased insulin sensitivity and glucose tolerance, and are resistant to high-fat diet-induced steatosis (62). Additionally, in the few studies that have been conducted, it appears that C2β generates PI3P, which is essential for receptor activation of various cell types, specifically, the proliferation of T-cells and activation of their TCRs (63, 64), B-cell receptor signaling (65), and most recently, mast cell activation through IgE receptor stimulation (66). Further, pharmacological inhibition of C2β is proposed to have therapeutic potential in diseases, which are IgE-associated (67). In the context of tuberculosis, however, the average downregulation of this gene across peripheral blood leukocytes in affected individuals suggests an overall dampening of the immune system. Future studies should assess the role of this protein during tuberculosis disease and whether PI3P intracellular levels are affected as this ultimately interferes with receptor activation and as a consequence, communication between the innate and adaptive immune systems.

## Concluding Remarks

Taken together, evidence from both mouse and human studies suggests a strong correlation between neutrophil hyper-reactivity in the tuberculosis-susceptible host. Early infection events indicate a higher-than-average influx of neutrophils to the infection site which appears to facilitate and promote *M. tb* growth (13). Evidence from transcriptomic studies indicate aberrations in gene expression of key molecules of the PI3-kinase pathway, as well as their regulators during tuberculosis disease. Notable roles of these molecules include modulating the IL-17/G-CSF axis, inducing leukocyte receptor activation, and regulating apoptosis and motility. Thus, the PI3–kinase pathway may play an especially prominent role in neutrophils of susceptible individuals and may be an intrinsic cause of tuberculosis disease severity and progression and, therefore, warrants further investigation.

## Author Contributions

The author confirms being the sole contributor of this work and approved it for publication.

## Conflict of Interest Statement

The author declares that the submitted work was carried out with no personal, professional, or financial relationships that could be construed as conflict of interest.
